# The role of NPC1L1 in cancer

**DOI:** 10.3389/fphar.2022.956619

**Published:** 2022-08-10

**Authors:** Renshuai Zhang, Jun Zeng, Wenjing Liu, Jingsen Meng, Chao Wang, Lingyu Shi, Shanbo Yang, Jing Chang, Dongming Xing

**Affiliations:** ^1^ Qingdao Cancer Institute, The Affiliated Hospital of Qingdao University, Qingdao University, Qingdao, China; ^2^ School of Basic Medicine, Qingdao University, Qingdao, China; ^3^ School of Life Sciences, Tsinghua University, Beijing, China

**Keywords:** NPC1L1, tumor, cholesterol, tumor therapy, cancer marker

## Abstract

Lipid metabolism appears to play significant roles in the development of cancer. Numerous studies have shown that the evolution of malignancies, including breast, prostate, and colorectal cancers, involves cholesterol in a profound manner. A crucial part in the intestinal absorption of cholesterol is played by Niemann–Pick C1-like 1 (NPC1L1), a cholesterol transporter protein that is widely expressed in the small intestine and liver. The importance of NPC1L1 in tumor prognosis has been demonstrated in investigations in the interim. NPC1L1 also has the potential to develop into a new therapeutic target and a cancer marker. There is, however, no comprehensive review that summarizes NPC1L1’s function in cancer. To this end, we outlined NPC1L1’s functions in carcinogenesis and treatment, along with resources that can be used to further comprehend the connection between NPC1L1 and tumors.

## 1 Introduction

The body needs cholesterol for many functions, but too much cholesterol can create hypercholesterolemia, which can cause atherosclerosis, stroke, and coronary heart disease (Alonso et al., 2013). Due to the additional ways in which cholesterol encourages cell division, invasion, and proliferation, it is crucial to the growth and development of tumors. Studies have revealed that the upregulation of the cholesterol synthesis level, the rise in cholesterol absorption, and the abnormal accumulation of a large number of metabolites are the main manifestations of the improper regulation of cholesterol metabolism in tumor cells. This results in improved tumor cell growth, survival, invasion, metastasis, and tumor microenvironment adaption. Tumor occurrence and growth are further encouraged (Yoshioka et al., 2000).

**FIGURE 1:**
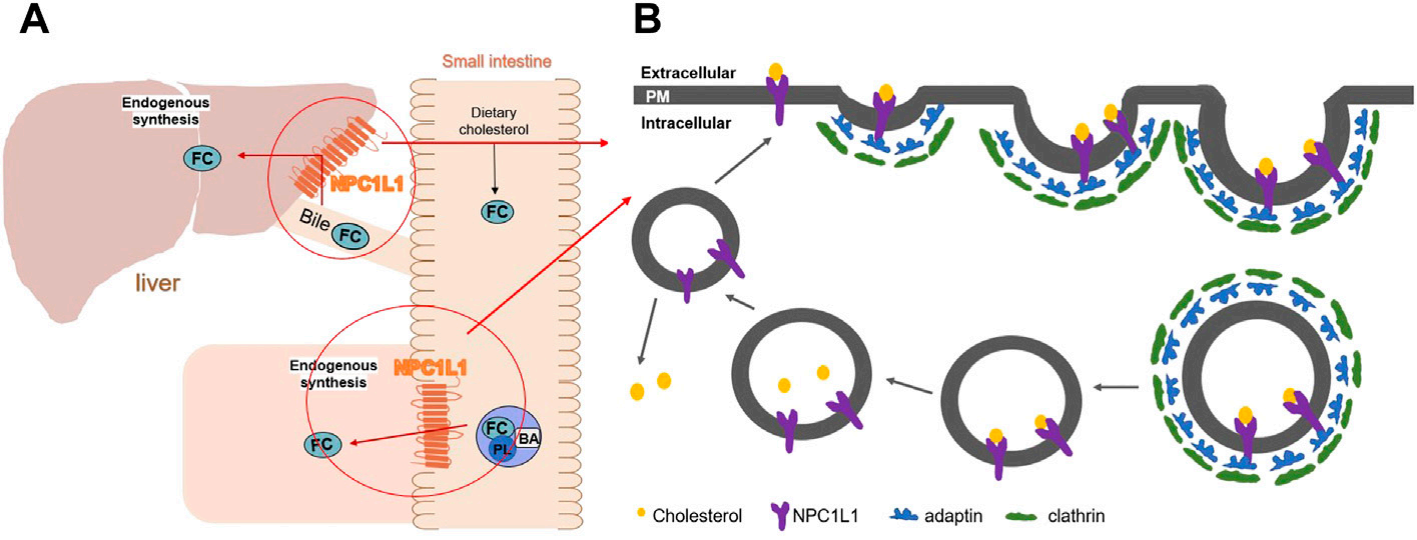
**(A)** Diagram of the mechanism of action of NPC1L1 in cholesterol transport in the small intestine and liver. **(B)** Working model for NPC1L1-mediated cholesterol uptake. (FC) Free cholesterol; (BA) bile acids; (PL) phospholipids; (PM) plasma membrane.

Niemann–Pick type C1-like 1 (NPC1L1) is a protein that is essential for intestinal cholesterol absorption and plays vital roles in dietary cholesterol absorption and biliary cholesterol resorption. With remarkable specificity, NPC1L1 mediates cholesterol entrance into small intestinal absorptive cells. A vesicular endocytosis process, as demonstrated by the studies, mediated cholesterol absorption by the NPC1L1 protein. When the extracellular cholesterol concentration was high, the plasma membrane protein NPC1L1 would endocytose the extracellular cholesterol and transfer it to the endocytic cycle (Ge et al., 2008). (Figure 1) As a specific target of NPC1L1, ezetimibe is a small molecule compound that can effectively and specifically inhibit the absorption of intestinal cholesterol. It is a medication used to treat coronary heart disease and hypercholesterolemia. According to research, it can lower plasma cholesterol by 15–20% (Bays et al., 2001; Dujovne et al., 2002). It is also a medication used to treat dyslipidemia that does not respond to statin therapy (Betters and Yu, 2010).

Obesity, hyperlipidemia, lipid storage disorders, and vascular diseases are all brought on by an abnormal cholesterol metabolism. Changes in cholesterol metabolism can significantly impact how quickly cancer develops and spreads. The current research on the association between NPC1L1 and cancer in carcinogenesis and cancer therapy is of utmost importance since NPC1L1 is a crucial member of cholesterol in intestinal absorption. First, we detailed the structure, usage, and distribution of the NPC1L1 protein in this article. Then, using the studies that were accessible, we summarized the connection between NPC1L1 and cancer for the first time.

## 2 Structure and function of NPC1L1

### 2.1 Structure of NPC1L1

**FIGURE 2:**
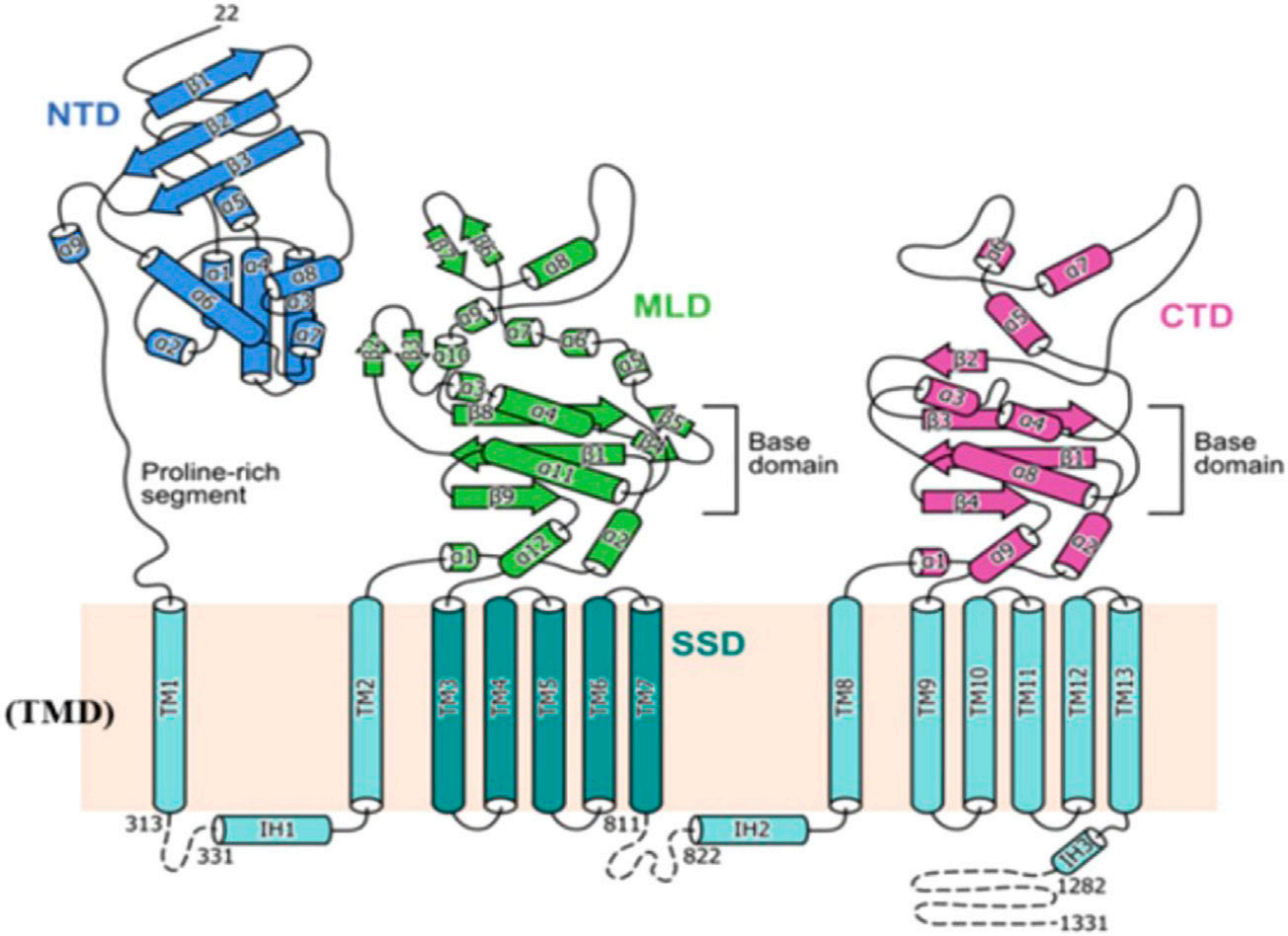
Topological representation of NPC1L1. NTD: N-terminal domain; TMD: transmembrane domain; MLD: middle luminal domain; CTD: C-terminal luminal domain; SSD: sterol-sensing domain (Huang et al., 2020).

NPC1L1 is a 1,332-amino acid membrane protein that is only found in primate hepatocyte tubular membranes and mammalian small intestinal brush membranes. The sequences of Niemann–Pick disease type C1 (NPC1) and NPC1L1 are comparable in 51 and 42 percent, respectively (Davies et al., 2000). A membrane protein called NPC1 performs a job in late endosomes and lysosomes (Pallottini and Pfrieger, 2020). As a homolog of NPC1, NPC1L1 likewise possesses a transmembrane domain with 13 cysteines and three major luminal structural domains in the extracellular area, the N-terminal domain (NTD), the middle domain (MLD), and the cysteine-rich domain (CTD). Thirteen molecules of membrane-embedded transmembrane helices (TM) make up the transmembrane domain (TMD) (Hu et al., 2021). The SSD domain is widely distributed in several regulatory protein substructures that are closely related to cholesterol metabolisms, such as NPC1, sterol regulatory element-binding protein cleavage activating protein (SCAP), and hydroxymethyl glutaryl-CoA reductase (HMG-CoA reductase) (Yang et al., 2012). (Figure 2)

### 2.2 Distribution of NPC1L1

NPC1L1 is tissue-specific in its distribution and is highly expressed in tissues connected to the digestive system. Currently, NPC1L1 is being investigated more in rodents and humans, and it has been discovered that there are small species differences in the distribution of NPC1L1 *in vivo*. The human liver and small intestine are the tissues with the highest levels of the NPC1L1 expression, followed by the stomach, ovary, lung tissues, and even minimal amounts in the brain tissues (Davies et al., 2000). However, the NPC1L1 expression in all tissues is less than 10% of that in the intestine (Davies et al., 2000). Further studies showed that NPC1L1 in small intestine tissues was mainly located in jejunal epithelial cells rather than in ileal epithelial cells. It was previously thought that the NPC1L1 protein was only expressed in cell membranes, especially in the cell membranes of the epithelial layer of the intestinal villi folds. Moreover, it was shown that NPC1L1 was dispersed in the cytoplasm and on the cell membrane (Yu et al., 2006). In rodents, NPC1L1 is highly expressed, especially in the small intestine, with minimal expression in liver tissues. Additionally, mouse testes and gallbladder tissues also showed high levels of the NPC1L1 expression (Cui et al., 2010; Wang and Song, 2012). The variety of NPC1L1 distribution among species is currently thought to be related to the degree of species evolution or possibly the outcome of long-term selection as a result of various survival settings. Cryo-EM structures of NPC1L1 were reported sequentially by three distinct research teams in the years 2020 and 2021. C.S. Huang et al. studied the structure of NPC1L1 derived from *Rattus*. Miao Qinghu et al. (Hu et al., 2021) and Long et al. (2021) studied the structure of human-derived NPC1L1. Their findings demonstrated a general similarity between the three-dimensional architecture of mouse and human NPC1L1.

### 2.3 Functions of NPC1L1

In humans, cholesterol is absorbed mainly in the proximal jejunum of the small intestine, where both dietary and biliary cholesterol types are absorbed (Reboul et al., 2012). Clinical investigations have demonstrated decreased membrane absorption and the subsequent transport of various lipids, as well as a 69 percent reduction in cholesterol uptake in NPC1L1-knockout mice (Iyer et al., 2005). Mice lacking NPC1L1 are resistant to hypercholesterolemia induced by a high-fat diet. Another study indicated that normal mice did not experience any significant changes in NPC1L1 or an increase in cholesterol absorption when bile acid and bile salt secretion and excretion were reduced while consuming a high-cholesterol diet (Alrefai et al., 2007). It is hypothesized that NPC1L1 may only work in the presence of diets high in cholesterol and bile acid salts.

### 3 Tumor suppression by NPC1L1 in cancer

#### 3.1 Colorectal cancer

Colorectal cancer (CRC) is a malignant tumor of the colon and rectum. It is also the third most common cancer and the fourth leading cause of cancer-related deaths worldwide (Brenner et al., 2014). Due to the changes in dietary habits, the incidence of CRC is rapidly increasing in many countries, including those in Eastern Europe, South America, and Asia (Arnold et al., 2017). Age, family history, inflammatory bowel disease, hereditary colorectal cancer, obesity, and diabetes are known as the risk factors for colorectal cancer.

**FIGURE 3:**
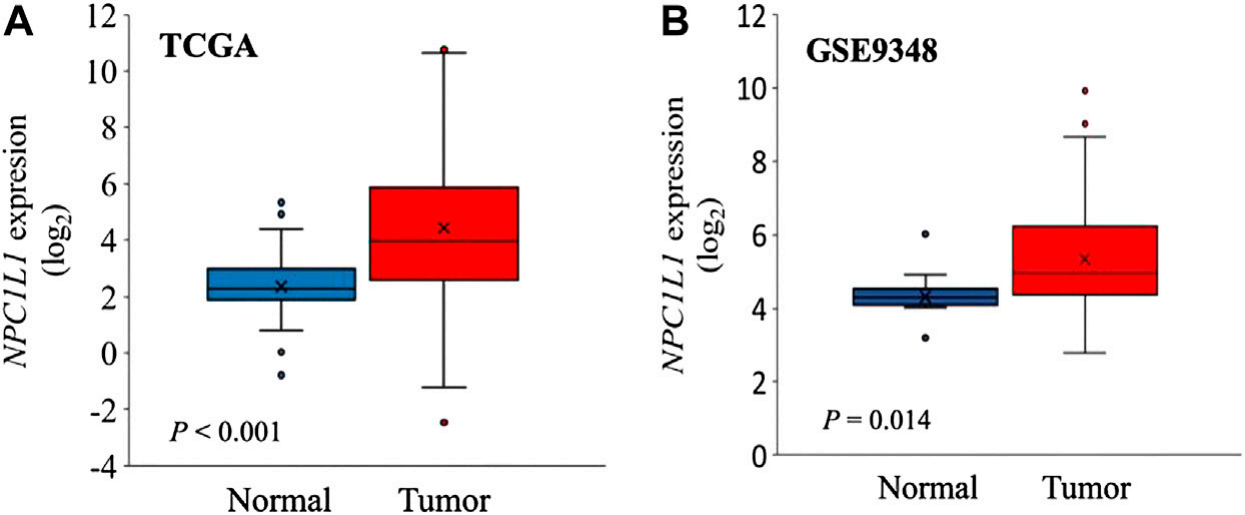
NPC1L1 expression in normal and CRC tissues. **(A)** NPC1L1 expression is higher in most CRC tissues than in normal tissues. The mean value of the NPC1L1 expression in normal tissues (blue box) is 7.00, and in CRC tissues (red box), it is 130.09. **(B)** Mean value of the NPC1L1 expression in normal tissues (blue box) is 22.69, and in CRC tissues (red box), it is 81.35 compared to normal tissues (GSE9348) (Kwon et al., 2021).

**FIGURE 4:**
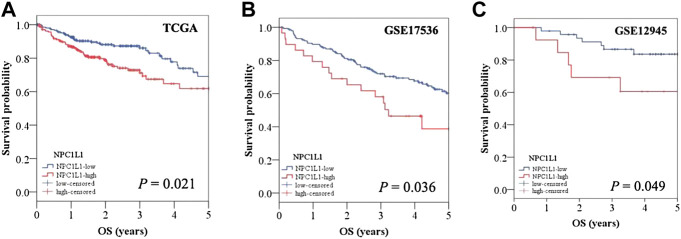
OS of CRC patients with a high NPC1L1 expression was significantly lower than that of CRC patients with a low NPC1L1 expression. The results of the two datasets (GSE17536 and GSE129451) collated in the CRC patients stratified into NPC1L1-low and NPC1L1-high groups also showed results consistent with those obtained from the analysis of TCGA dataset (Kwon et al., 2021).

**FIGURE 5:**
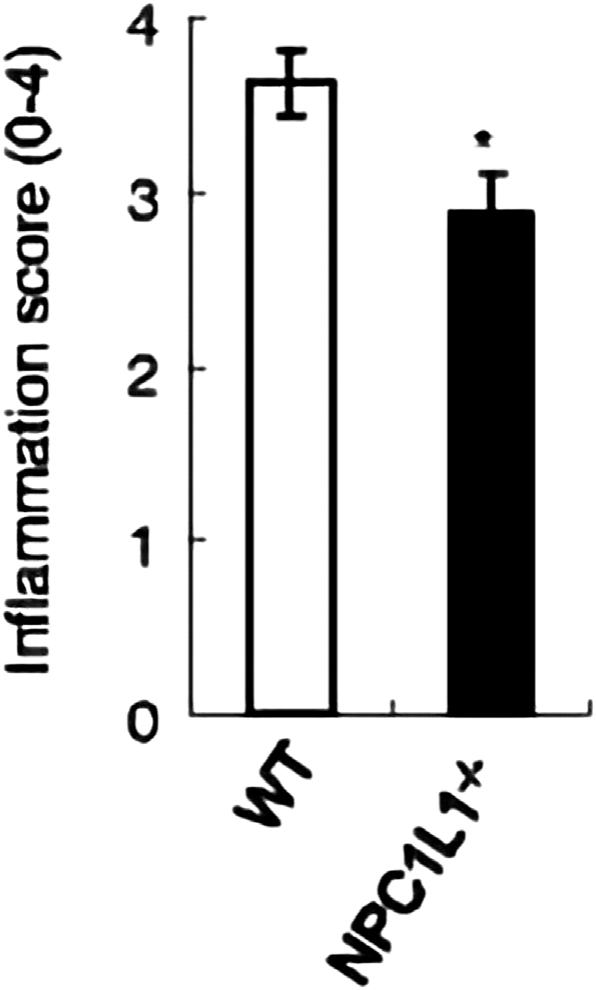
NPC1L1-knockout mice have significantly lower intestinal inflammation scores than wild-type mice (He et al., 2015).

**FIGURE 6:**
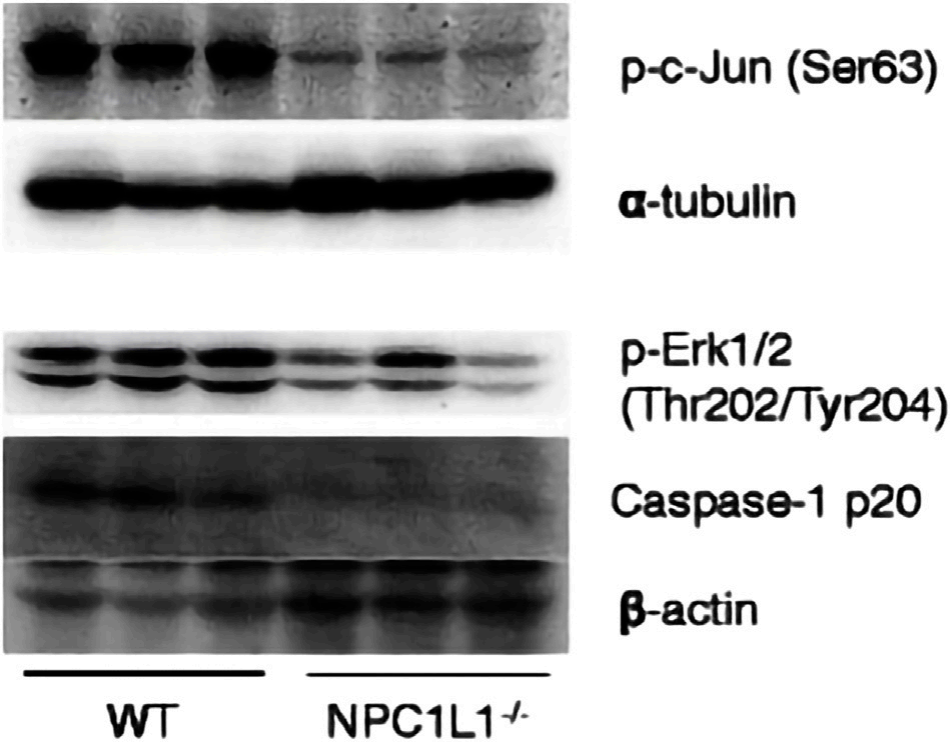
pc-Jun, p-ERK, and caspase-1 p20 protein expressions in tumors measured by protein blotting. pc-Jun, p-ERK and caspase-1 p20 were significantly reduced by NPC1L1 knockdown. pc-Jun, p-ERK, and caspase-1 p20 were also reduced by NPC1L1 knockdown (He et al., 2015).

The development and prognosis of colorectal cancer have been shown to be associated with an altered lipid metabolism. High cholesterol intake is associated with an increased risk of colorectal cancer. Elevated serum cholesterol levels are associated with the risk of developing colorectal cancer (Jarvinen et al., 2001). Ryuk et al. (Kwon et al., 2021) explored whether alterations in the NPC1L1 expression are associated with the development and prognosis of human colorectal cancer. In comparison to normal tissues, CRC tissues showed considerably greater levels of the NPC1L1 expression (normal: mean 7.00, CRC: mean 130.09, and *p* 0.05) (Figure 3). This study determined whether the NPC1L1 expression had an impact on CRC patients’ prognoses. NPC1L1 has an impact on colorectal cancer patients’ overall survival, with patients in the NPC1L1 high-expression group having a worse OS than NPC1L1 patients, according to a KM analysis of the NPC1L1 low-expression group and the NPC1L1 high-expression group on OS (Figure 4). It was revealed that NPC1L1 has value as a standalone prognostic factor for colorectal cancer, and it was found that the NPC1L1 expression was highly correlated with the prognosis of the disease. NPC1L1, along with other known prognostic markers, can be independent prognostic markers for colorectal cancer. Jianming H et al. (He et al., 2015) explored the role of NPC1L1 in colorectal carcinogenesis *in vivo* using transgenic mice. Their findings have shown that NPC1L1 deletion in mice reduced carcinogenesis linked to colitis. Although only expressed in the small intestine of mice, NPC1L1 mRNA was substantially elevated in the liver and small intestine of humans. Its mRNA was also present in the colon but at a very low level (Chen et al., 2018). Therefore, it is unlikely that the NPC1L1 knockdown inhibits the growth of malignancies linked to colitis. In the small intestine and liver, NPC1L1 primarily functions as a sterol transporter protein that controls lipid homeostasis. Plasma lipids, particularly cholesterol, are closely linked to colon cancer and, through inflammation, cause animals to develop tumors associated with colitis (Vinson et al., 2016). Cholesterol was evidently decreased by NPC1L1 knockdown. Additionally, the inflammatory markers pc-Jun, p-ERK, and caspase-1 p20 in colorectal cancers were considerably decreased by NPC1L1 knockdown (Figures 5, 6). Therefore, NPC1L1 knockdown decreases carcinogenesis associated with colitis, which may be brought on by the decrease in plasma lipids, particularly cholesterol, brought on by its knockdown, which lessens the sensitivity to inflammatory stimuli.

### 3.2 Head and neck squamous cell carcinoma

With more than 550,000 new cases and 300,000 fatalities each year, head and neck squamous cell carcinoma (HNSCC), which comprises oral cavity cancer (OC) and oropharyngeal cancer (OPC), is the sixth most prevalent cancer worldwide (Saba et al., 2011). Despite advances in HNSCC treatment, the 10-year survival rate only varies from 19 to 59 percent, and recurrence rates remain significant. Patients frequently endure a combination of radiation, chemotherapy, and surgery, which can cause severe morbidity. Smoking, drinking, and the human papillomavirus (HPV), which is primarily linked to oropharyngeal cancer (Gormley et al., 2021), have all been identified as risk factors (Warnakulasuriya, 2009).

According to several observational studies, statin use is associated negatively with cancer survival and HNSCC risk. Other research studies, however, have shown scant evidence of any impact. Using two-sample Mendelian randomization (MR), the relationship between the targets of genetically proxied cholesterol-lowering drugs and other circulating lipid profiles with OC and OPC risks was evaluated. Germline genetic variations in the genes for HMGCR, NPC1L1, CETP, PCSK9, and LDLR were utilized to simulate the effects of low-density lipoprotein cholesterol-lowering treatments in the initial analysis. There is scant evidence that inherited NPC1L1 increases the chance of developing OC and OPC (Gormley et al., 2021).

### 3.3 Ovarian cancer

One of the world’s most dangerous malignant tumors is ovarian cancer due to the early stages of ovarian cancer’s lack of visible signs. The so-called “silent killer” is frequently identified in advanced stages in patients. Based on the genetic alterations and the cell shape of epithelial ovarian cancer, type I and type II ovarian tumors can be distinguished. Low-grade plasmacytomas, endometrioid carcinomas, clear cell carcinomas, and mucinous carcinomas are examples of type I tumors. High-grade plasmacytomas and undifferentiated carcinomas are examples of type II tumors (He et al., 2021). High-grade plasmacytomas and undifferentiated carcinomas are the two main type II tumors.

When the analysis was limited to the general population or BRCA1/2 mutation carriers, there was no significant association between genetically close NPC1L1 or PCSK9 inhibition or low-density lipoprotein cholesterol levels and epithelial ovarian cancer in the Mendelian randomized analysis of 22,406 women with invasive epithelial ovarian cancer and 40,941 control individuals (Yarmolinsky et al., 2020). It is possible that circulating cholesterol is not the cause of the observed relationship between HMG-CoA reductase inhibition and ovarian cancer due to the lack of genetically close inhibition between NPC1L1 and PCSK9 inhibition and genetically close LDL cholesterol levels (Yarmolinsky et al., 2020).

### 3.4 Hepatocellular carcinoma

Liver cancer accounts for 8.2% of all cancer deaths globally, ranking sixth in cancer incidence. Additionally, it ranks third in the world for cancer-related fatalities (Siegel, Miller, and Jemal). Intrahepatic cholangiocarcinoma, hepatocellular carcinoma (HCC), fibrous lamellar carcinoma, and hepatoblastoma are several types of primary liver cancer. These classifications differ significantly in terms of their molecular, histological, and pathological traits. Of the instances of liver cancer, 85 to 90 percent are caused by HCC alone (Sia et al., 2017). Tumor removal, liver transplantation, and ablation are all forms of treatment for HCC (Llovet et al., 2004). Tumor removal, liver transplantation, and ablation are all forms of treatment for HCC. Only patients with early disease diagnoses can, however, get this treatment. Additionally, recent research has revealed that only 20% of patients receive an early diagnosis (Farinati et al., 2009).

**FIGURE 7:**
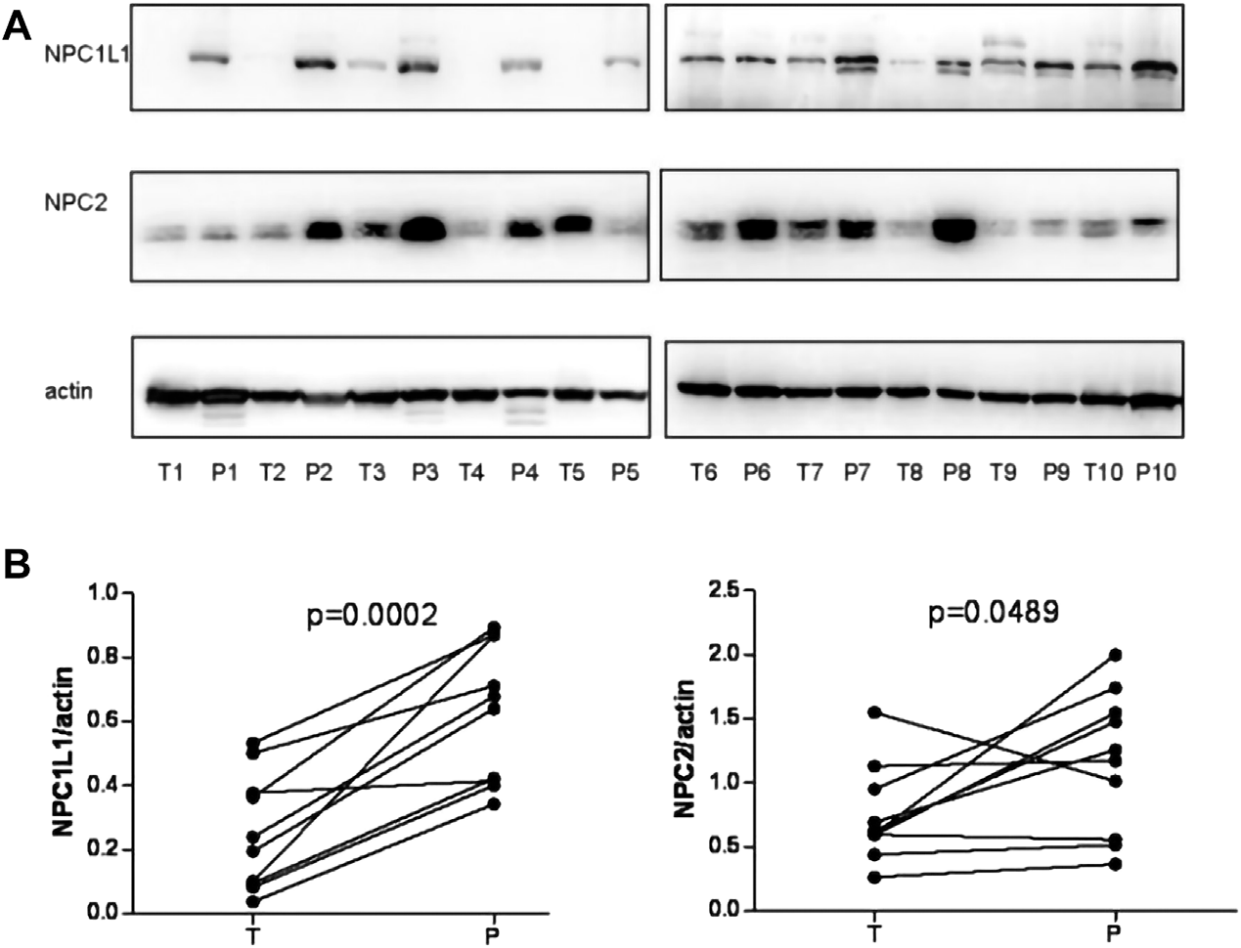
**(A)** NPC1L1 and NPC2 expressions were detected in 10 pairs of HCC tissues (T) and peritumoral tissues (*p*) by Western blot analysis. The expressions of NPC1L1 and NPC2 were significantly decreased in HCC tissues. Meanwhile, the mRNA expressions of NPC1L1 and NPC2 expression levels were also decreased in HCC tissues compared with peritumoral tissues. **(B)** Quantitation of proteins from Western blot analyses shows that both NPC1L1 and NPC2 expressions were significantly reduced in the HCC tissue (T) (Chen et al., 2018).

**FIGURE 8:**
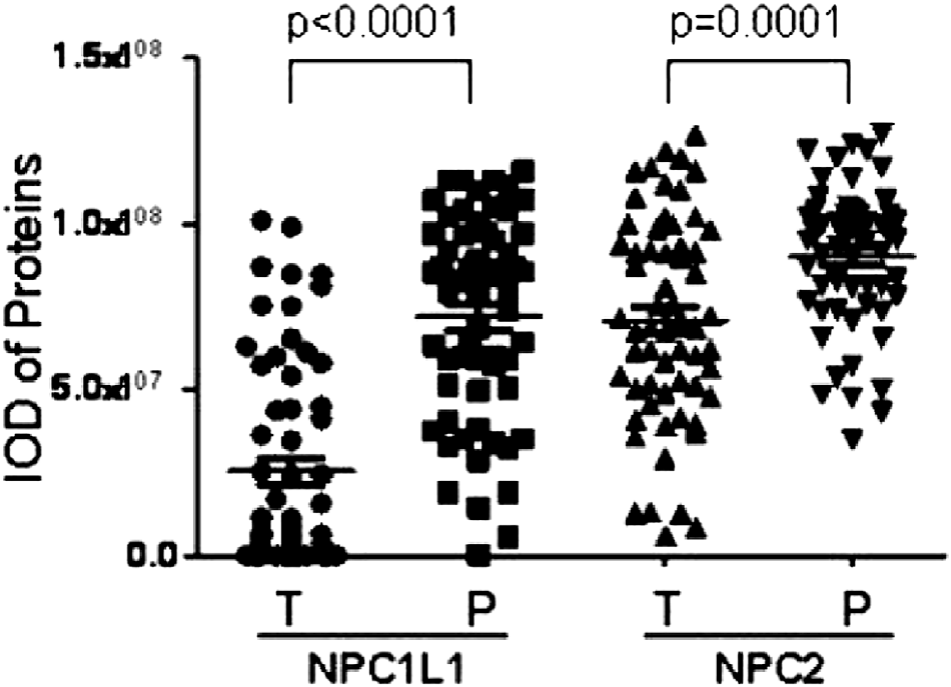
Scatter plot showing paired peritumor tissues shows that NPC1L1 and NPC2 expressions in HCC tumor tissues are usually lower than those in peritumor tissues (NPC1L1 *p* < 0.0001; NPC2 *p* = 0.0001) (Chen et al., 2018).

Chen et al. (2018) investigated the prognostic value of NPC1L1 in human primary hepatocellular carcinoma (HCC). According to the findings, NPC1L1 and NPC2 are not as highly expressed in the HCC liver tissue as in the peritumoral liver tissue. There is also less NPC1L1 mRNA expression in the HCC tissue than in the peritumoral tissue (Figure 7). NPC1L1 inhibition has previously been demonstrated to protect against metabolic diseases such as fatty liver disease, obesity, diabetes, and atherosclerosis (Park, 2013). This is the first study to discuss NPC1L1’s function in HCC. When matched peritumoral liver tissues were compared to HCC, NPC1L1 was shown to be lower in the latter. In postoperative HCC patients, a low NPC1L1 protein expression may be a predictor of worse OS and TTR. The expressions of NPC1L1 and NPC2 in HCC tumor tissues were often lower than those in peritumor tissues, according to the patterns of expression in tumor and peritumor tissues (Figure 8). The NPC1L1/NPC2 combination was also discovered as a separate prognostic factor for OS and TTR in postoperative HCC patients, and this study was the first to reveal the prognostic usefulness of NPC1L1 in HCC. Therefore, determining the levels of NPC1L1 and NPC2 expressions in the tissues of HCC patients may give doctors information about the risk of postoperative OS and TTR in HCC patients and may aid in the investigation of the mechanism behind the association between cholesterol and HCC disease.

### 3.5 Pancreatic cancer

As one of the 14 most prevalent malignancies worldwide, pancreatic cancer is the seventh most common cause of cancer-related deaths (Rawla et al., 2019). Pancreatic cancer often has a terrible prognosis, with a 5-year survival rate of fewer than 5% on average (Carter et al., 2021). More than 90% of the instances of pancreatic cancer are pancreatic ductal adenocarcinoma (PDAC). It is distinguished by an aberrant expression of mucin and nucleoside transporter proteins, as well as extensive pro-fibroproliferative stromal growth near the malignant tissue (Murthy et al., 2018). As antecedents of PDAC, three different pancreatic ductal precancerous lesions—pancreatic intraepithelial neoplasia, mucinous cystic neoplasm, and intraductal papillary mucinous neoplasm—have been found (Suh et al., 2017). The respectability of the main tumor—defined as the lack of distant metastases and topographical contacts that permit R0 resection—determines the pancreatic cancer treatment plans. In addition to chemotherapy, surgical resection is the primary treatment option for individuals who are suitable for it for curable pancreatic cancer. However, around 90% of patients have cancers that are either locally progressed or metastatic at the time of diagnosis, making them unsuitable for surgical excision. Jaundice treatment, systemic control, palliative radiation, and chemotherapy are the sole alternatives for these individuals.

**FIGURE 9:**
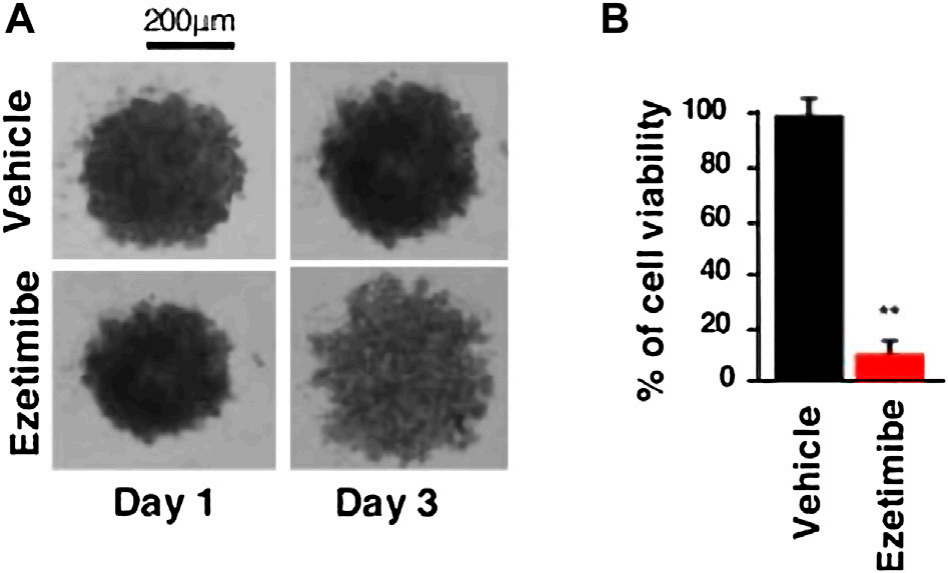
**(A)** Representative image of the PDAC012T-derived spheroids treated with ezetimibe (50 µM) or vehicle after 3 days. **(B)** Cell viability of spheroids was measured by CellTiter-Glo and expressed as a percentage of the vehicle-treated spheroids (∗∗*p* < 0.01) (Nicolle et al., 2017).

Guillaumond et al. (2015) proposed that an abnormal cholesterol uptake is associated with the proliferation and survival of pancreatic cancer cells. In contrast, an overexpression of the intestinal cholesterol uptake regulator NPC1L1 is associated with extensive hypomethylation. Ezetimibe, a clinically available drug approved for FAD, is a competitor to cholesterol. Nicolle et al. (2017) investigated the specificity of NPC1L1 inhibition on PDAC survival. The outcomes demonstrated a significant impact of ezetimibe therapy on PDAC growth, following NPC1L1 knockdown, including modifications in cell viability and volume (Figure 9). It showed that NPC1L1 is a productive therapeutic target for the ezetimibe therapy of PDAC. Additionally, their findings showed that ezetimibe treatment had no impact on gemcitabine’s cytotoxicity, indicating that patients could get treated with NPC1L1 transporter protein inhibitors in addition to traditional anti-cancer medications without jeopardizing the efficacy of either. Preclinical studies have demonstrated, among other things, that the use of the particular inhibitor ezetimibe or a deletion strategy dramatically impacted PDAC survivability. These lend credence to the idea that NPC1L1 might work well as a therapeutic target for pancreatic cancer.

## 4 Conclusion and outlook

Cholesterol aids in the proliferation, invasion, and subduction of cells and is crucial for the development and growth of tumors. As a crucial protein in intestinal cholesterol absorption, NPC1L1 plays vital functions in the development and spread of tumors. The most recent research indicates that NPC1L1 can be employed as a standalone prognostic marker in colorectal cancer. Additionally, it proved to be a reliable prognostic factor for hepatocellular carcinoma. Additionally, NPC1L1 may be a valuable therapeutic target for pancreatic cancer. The association between NPC1L1 and other malignancies has not been proven by recent investigations. The significance of NPC1L1 in carcinogenesis and cancer therapy, however, merits additional research to offer new therapeutic avenues for clinical cancers due to the role of cholesterol in tumorigenesis and development.

Overall, the current studies on the connection between NPC1L1 and cancers are deficient, however, due to the function of NPC1L1 in cholesterol uptake and the link between cholesterol and cancer. Future research examining this connection might reveal other targets to impede the progression of cholesterol-dependent cancer.

## References

[B1] Alonso R. Mata P. Zambon D. Mata N. Fuentes-Jimenez F. (2013). Early diagnosis and treatment of familial hypercholesterolemia: Improving patient outcomes. Expert Rev. cardiovasc. Ther. 11 (3), 327–342. 10.1586/erc.13.7 23469913

[B2] Alrefai W. A. Annaba F. Sarwar Z. Dwivedi A. Saksena S. Singla A. (2007). Modulation of human Niemann-Pick C1-like 1 gene expression by sterol: Role of sterol regulatory element binding protein 2. Am. J. Physiol. Gastrointest. Liver Physiol. 292 (1), G369–G376. 10.1152/ajpgi.00306.2006 17008555

[B3] Arnold M. Sierra M. S. Laversanne M. Soerjomataram I. Jemal A. Bray F. (2017). Global patterns and trends in colorectal cancer incidence and mortality. Gut 66 (4), 683–691. 10.1136/gutjnl-2015-310912 26818619

[B4] Bays H. E. Moore P. B. Drehobl M. A. Rosenblatt S. Toth P. D. Dujovne C. A. (2001). Effectiveness and tolerability of ezetimibe in patients with primary hypercholesterolemia: Pooled analysis of two phase II studies. Clin. Ther. 23 (8), 1209–1230. 10.1016/s0149-2918(01)80102-8 11558859

[B5] Betters J. L. Yu L. (2010). NPC1L1 and cholesterol transport. FEBS Lett. 584 (13), 2740–2747. 10.1016/j.febslet.2010.03.030 20307540 PMC2909875

[B6] Brenner H. Kloor M. Pox C. P. (2014). Colorectal cancer. Lancet 383 (9927), 1490–1502. 10.1016/S0140-6736(13)61649-9 24225001

[B7] Carter C. J. Mekkawy A. H. Morris D. L. (2021). Role of human nucleoside transporters in pancreatic cancer and chemoresistance. World J. Gastroenterol. 27 (40), 6844–6860. 10.3748/wjg.v27.i40.6844 34790010 PMC8567477

[B8] Chen K. J. Jin R. M. Shi C. C. Ge R. L. Hu L. Zou Q. F. (2018). The prognostic value of Niemann-Pick C1-like protein 1 and Niemann-Pick disease type C2 in hepatocellular carcinoma. J. Cancer 9 (3), 556–563. 10.7150/jca.19996 29483961 PMC5820923

[B9] Cui W. Jiang Z. Y. Cai Q. Zhang R. Y. Wu W. Z. Wang J. C. (2010). Decreased NPC1L1 expression in the liver from Chinese female gallstone patients. Lipids Health Dis. 9, 17. 10.1186/1476-511X-9-17 20144195 PMC2841174

[B10] Davies J. P. Levy B. Ioannou Y. A. (2000). Evidence for a niemann-pick C (NPC) gene family: Identification and characterization of NPC1L1. Genomics 65 (2), 137–145. 10.1006/geno.2000.6151 10783261

[B11] Dujovne C. A. Ettinger M. P. McNeer J. F. Lipka L. J. LeBeaut A. P. Suresh R. (2002). Efficacy and safety of a potent new selective cholesterol absorption inhibitor, ezetimibe, in patients with primary hypercholesterolemia. Am. J. Cardiol. 90 (10), 1092–1097. 10.1016/s0002-9149(02)02798-4 12423709

[B12] Farinati F. Sergio A. Baldan A. Giacomin A. Di Nolfo M. A. Del Poggio P. (2009). Early and very early hepatocellular carcinoma: When and how much do staging and choice of treatment really matter? A multi-center study. BMC Cancer 9, 33. 10.1186/1471-2407-9-33 19171074 PMC2640412

[B13] Ge L. Wang J. Qi W. Miao H. H. Cao J. Qu Y. X. (2008). The cholesterol absorption inhibitor ezetimibe acts by blocking the sterol-induced internalization of NPC1L1. Cell Metab. 7 (6), 508–519. 10.1016/j.cmet.2008.04.001 18522832

[B14] Gormley M. Yarmolinsky J. Dudding T. Burrows K. Martin R. M. Thomas S. (2021). Using genetic variants to evaluate the causal effect of cholesterol lowering on head and neck cancer risk: A mendelian randomization study. PLoS Genet. 17 (4), e1009525. 10.1371/journal.pgen.1009525 33886544 PMC8096036

[B15] Guillaumond F. Bidaut G. Ouaissi M. Servais S. Gouirand V. Olivares O. (2015). Cholesterol uptake disruption, in association with chemotherapy, is a promising combined metabolic therapy for pancreatic adenocarcinoma. Proc. Natl. Acad. Sci. U. S. A. 112 (8), 2473–2478. 10.1073/pnas.1421601112 25675507 PMC4345573

[B16] He J. Shin H. Wei X. Kadegowda A. K. Chen R. Xie S. K. (2015). NPC1L1 knockout protects against colitis-associated tumorigenesis in mice. BMC Cancer 15, 189. 10.1186/s12885-015-1230-0 25881076 PMC4378275

[B17] He J. Siu M. K. Y. Ngan H. Y. S. Chan K. K. L. (2021). Aberrant cholesterol metabolism in ovarian cancer: Identification of novel therapeutic targets. Front. Oncol. 11, 738177. 10.3389/fonc.2021.738177 34820325 PMC8606538

[B18] Hu M. Yang F. Huang Y. You X. Liu D. Sun S. (2021). Structural insights into the mechanism of human NPC1L1-mediated cholesterol uptake. Sci. Adv. 7 (29), eabg3188. 10.1126/sciadv.abg3188 34272236 PMC8284890

[B19] Huang C. S. Yu X. Fordstrom P. Choi K. Chung B. C. Roh S. H. (2020). Cryo-EM structures of NPC1L1 reveal mechanisms of cholesterol transport and ezetimibe inhibition. Sci. Adv. 6 (25), eabb1989. 10.1126/sciadv.abb1989 32596471 PMC7304964

[B20] Iyer S. P. Yao X. Crona J. H. Hoos L. M. Tetzloff G. Davis H. R. Jr. (2005). Characterization of the putative native and recombinant rat sterol transporter Niemann-Pick C1 like 1 (NPC1L1) protein. Biochim. Biophys. Acta 1722 (3), 282–292. 10.1016/j.bbagen.2004.12.021 15777641

[B21] Jarvinen R. Knekt P. Hakulinen T. Rissanen H. Heliovaara M. (2001). Dietary fat, cholesterol and colorectal cancer in a prospective study. Br. J. Cancer 85 (3), 357–361. 10.1054/bjoc.2001.1906 11487265 PMC2364063

[B22] Kwon R. J. Park E. J. Lee S. Y. Lee Y. Hwang C. Kim C. (2021). Expression and prognostic significance of niemann-pick C1-like 1 in colorectal cancer: A retrospective cohort study. Lipids Health Dis. 20 (1), 104. 10.1186/s12944-021-01539-0 34511128 PMC8436523

[B23] Llovet J. M. Fuster J. Bruix J. Barcelona-Clinic Liver Cancer G. (2004). The barcelona approach: Diagnosis, staging, and treatment of hepatocellular carcinoma. Liver Transpl. 10 (2), S115–S120. 10.1002/lt.20034 14762851

[B24] Long T. Liu Y. Qin Y. DeBose-Boyd R. A. Li X. (2021). Structures of dimeric human NPC1L1 provide insight into mechanisms for cholesterol absorption. Sci. Adv. 7 (34), eabh3997. 10.1126/sciadv.abh3997 34407950 PMC8373123

[B25] Murthy D. Attri K. S. Singh P. K. (2018). Phosphoinositide 3-kinase signaling pathway in pancreatic ductal adenocarcinoma progression, pathogenesis, and therapeutics. Front. Physiol. 9, 335. 10.3389/fphys.2018.00335 29670543 PMC5893816

[B26] Nicolle R. Blum Y. Marisa L. Loncle C. Gayet O. Moutardier V. (2017). Pancreatic adenocarcinoma therapeutic targets revealed by tumor-stroma cross-talk analyses in patient-derived xenografts. Cell Rep. 21 (9), 2458–2470. 10.1016/j.celrep.2017.11.003 29186684 PMC6082139

[B27] Pallottini V. Pfrieger F. W. (2020). Understanding and treating niemann-pick type C disease: Models matter. Int. J. Mol. Sci. 21 (23), E8979. 10.3390/ijms21238979 33256121 PMC7730076

[B28] Park S. W. (2013). Intestinal and hepatic niemann-pick c1-like 1. Diabetes Metab. J. 37 (4), 240–248. 10.4093/dmj.2013.37.4.240 23991401 PMC3753488

[B29] Rawla P. Sunkara T. Gaduputi V. (2019). Epidemiology of pancreatic cancer: Global trends, etiology and risk factors. World J. Oncol. 10 (1), 10–27. 10.14740/wjon1166 30834048 PMC6396775

[B30] Reboul E. Soayfane Z. Goncalves A. Cantiello M. Bott R. Nauze M. (2012). Respective contributions of intestinal niemann-pick C1-like 1 and scavenger receptor class B type I to cholesterol and tocopherol uptake: *In vivo* v. *in vitro* studies. Br. J. Nutr. 107 (9), 1296–1304. 10.1017/S0007114511004405 21929836

[B31] Saba N. F. Goodman M. Ward K. Flowers C. Ramalingam S. Owonikoko T. (2011). Gender and ethnic disparities in incidence and survival of squamous cell carcinoma of the oral tongue, base of tongue, and tonsils: A surveillance, epidemiology and end results program-based analysis. Oncology 81 (1), 12–20. 10.1159/000330807 21912193 PMC3186716

[B32] Sia D. Villanueva A. Friedman S. L. Llovet J. M. (2017). Liver cancer cell of origin, molecular class, and effects on patient prognosis. Gastroenterology 152 (4), 745–761. 10.1053/j.gastro.2016.11.048 28043904 PMC12160040

[B33] Siegel R. L. Miller K. D. Jemal A. (202). Cancer statistics, 2020. Ca. Cancer J. Clin. 70 (1), 7–30. 10.3322/caac.21590 31912902

[B34] Suh H. Pillai K. Morris D. L. (2017). Mucins in pancreatic cancer: Biological role, implications in carcinogenesis and applications in diagnosis and therapy. Am. J. Cancer Res. 7 (6), 1372–1383. 28670497 PMC5489784

[B35] Vinson K. E. George D. C. Fender A. W. Bertrand F. E. Sigounas G. (2016). The Notch pathway in colorectal cancer. Int. J. Cancer 138 (8), 1835–1842. 10.1002/ijc.29800 26264352

[B36] Wang L. J. Song B. L. (2012). Niemann-Pick C1-Like 1 and cholesterol uptake. Biochim. Biophys. Acta 1821 (7), 964–972. 10.1016/j.bbalip.2012.03.004 22480541

[B37] Warnakulasuriya S. (2009). Global epidemiology of oral and oropharyngeal cancer. Oral Oncol. 45 (4-5), 309–316. 10.1016/j.oraloncology.2008.06.002 18804401

[B38] Yang J. Y. Hu Y. W. Zhang P. Zheng L. Wang Q. (2012). Progress of Niemann-Pick type C1 like 1 on cholesterol metabolism. Sheng Li Xue Bao 64 (6), 721–728. 23258338

[B39] Yarmolinsky J. Bull C. J. Vincent E. E. Robinson J. Walther A. Smith G. D. (2020). Association between genetically proxied inhibition of HMG-CoA reductase and epithelial ovarian cancer. JAMA 323 (7), 646–655. 10.1001/jama.2020.0150 32068819 PMC7042851

[B40] Yoshioka Y. Sasaki J. Yamamoto M. Saitoh K. Nakaya S. Kubokawa M. (2000). Quantitation by (1)H-NMR of dolichol, cholesterol and choline-containing lipids in extracts of normal and phathological thyroid tissue. NMR Biomed. 13 (7), 377–383. 10.1002/1099-1492(200011)13:7<377::aid-nbm658>3.0.co;2-e 11114060

[B41] Yu L. Bharadwaj S. Brown J. M. Ma Y. Du W. Davis M. A. (2006). Cholesterol-regulated translocation of NPC1L1 to the cell surface facilitates free cholesterol uptake. J. Biol. Chem. 281 (10), 6616–6624. 10.1074/jbc.M511123200 16407187

